# Factors influencing caster board skill acquisition

**DOI:** 10.3389/fpsyg.2025.1643100

**Published:** 2025-11-07

**Authors:** Hiroo Suzuki, Takehito Hirakawa, Yuji Yamamoto

**Affiliations:** 1 Faculty of Economics, Ryukoku University, Kyoto, Japan; 2 School of Health and Sport Sciences, Osaka University of Health and Sport Sciences, Osaka, Japan; 3 Department of Psychological Sciences, Niigata University of Health and Welfare, Niigata, Japan

**Keywords:** motor learning, longitudinal observation, individual differences, variability of movement, dynamical systems theory

## Abstract

In motor learning research, various whole-body movement tasks have been examined using a dynamical systems approach. Prior studies have highlighted that differences in learning strategies and variability in movement contribute to individual differences in motor learning. Building on these findings, this study investigated the learning process of seven beginners as they attempted to ride a caster board for the first time and progressed until they were able to stand and ride it. Specifically, we aimed to compare and contrast commonalities and differences in the learning process to identify the factors contributing to individual differences and to clarify the motor skills crucial for mastering the caster board. To quantify movement changes associated with learning, we analyzed the initial velocity of the board and the amplitude of trunk rotational movement. Trial-by-trial changes were calculated to determine which variable exhibited greater change for each participant. Across all the participants, both initial velocity and trunk rotational movement increased with practice. These findings suggest that accelerating the board's initial velocity, which enhances stability, and increasing the amplitude of trunk rotational movement, which generates propulsive force, are both critical for mastering the caster board riding. However, the number of trials required to achieve the learning task varied by more than 100 trials across participants, and individual differences were also evident in the movement patterns at task completion. Case-based analyses revealed that these differences were influenced by the movement patterns performed in the early trials and by the variability in movement patterns executed across trials.

## 1 Introduction

Sports instructors frequently observe that, even when practicing in the same environment, some learners achieve proficiency quickly while others do not. Specifically, both the mastery of particular motor tasks and the rate of learning (how quickly a task is accomplished) vary among learners.

Motor control theory, motor learning theory, and theories of teaching movement are closely related but conceptually distinct: Motor control focuses on the mechanisms by which the brain and nervous system coordinate muscles and joints to produce purposeful movement; motor learning examines how movements change through practice and experience, and teaching theory applies these insights in instructional contexts (Schmidt et al., 2019). Moreover, motor learning theory has traditionally been developed through laboratory-based tasks such as handwriting (Teulings et al., 1983) and ball throwing (Kerr and Booth, 1978), building on closed-loop theory (Adams, 1971) and schema theory (Schmidt, 1975). It has further evolved from computational neuroscience to address problems like arm trajectory generation in movements such as reaching (Kawato, 1999). However, the control and learning processes of whole-body movements in sports cannot be fully elucidated using information processing or computational approaches. This is because controlling the numerous muscles and joints involved in whole-body movement requires solving the “degree of freedom” problem (Bernstein, 1967). In this regard, the dynamical systems approach offers an integrative framework that addresses control, learning, and teaching together.

Within the framework of the dynamical systems approach, behavioral changes during motor learning are conceptualized as self-organization processes in which the body overcomes redundant degrees of freedom and develops control. From this perspective, diverse motor learning processes have been investigated (Davids et al., 2003). Notably, this approach integrates Bernstein's concept of coordinative structures, the ecological perception theory of Gibson (1979), and Turvey' (1990) emphasis on perception-action dynamics. Consequently, motor learning is understood as a process through which the system acquires new, stable coordinative structures (i.e., new attractors) within a dynamical system (Haken et al., 1985; Beek et al., 1995; Warren, 2006). Studies adopting this perspective have examined movements such as ski slalom (van Emmerik et al., 1989; Vereijken et al., 1992), juggling (Beek and van Santvoord, 1992), and soccer kicking (Hodges et al., 2005).

The acquisition of new coordinative structures in motor learning exhibits individual differences owing to various factors (Zanone and Kelso, 1992; Ackerman, 2014). One such factor is intrinsic dynamics (Newell et al., 2001; Chow et al., 2016), which reflect coordinative structures shaped by prior motor experiences. Intrinsic dynamics strongly influence the acquisition of new coordinative structures (Schöner, 1989). For example, a learner with baseball experience may more readily acquire the tennis service motion due to the similarity in throwing mechanics (Reid et al., 2015). Thus, intrinsic dynamics are thought to underlie individual differences in motor learning. Additionally, the movement patterns exhibited during early learning trials influence the coordinative structures observed upon task completion (Kostrubiec et al., 2012; King et al., 2012). For example, in three-ball cascade juggling, two stable coordinative structures are observed, defined by three variables: ball flight time, the interval between grasping and throwing, and the time during which the hand is unloaded. Distinct learning pathways emerge depending on how closely a learner's initial movement patterns align with these coordination structures (Beek and van Santvoord, 1992; Hashizume and Matsuo, 2004; Yamamoto et al., 2015). Additionally, individual differences in learning rate are influenced by movement variability during the learning process. In particular, Wu et al. (2014) found that greater baseline movement variability (i.e., before learning) was linked to a faster subsequent learning rate during a motor task that involved drawing a novel figure using a mirror. This finding suggests that movement variability can promote motor learning.

Based on these findings, this study aims to clarify factors related to individual differences in motor learning from the perspective of the dynamical systems approach. To achieve this, it is necessary to compare learners who require different amounts of practice to achieve a clearly defined learning task. We, therefore, selected the caster board, a task that highlights individual differences in learning rate, for longitudinal observation.

A caster board, also known as an Essboard or waveboard, resembles a skateboard. It consists of two rotating decks connected by a torsion bar, with a caster attached to the bottom of each deck. Forward motion is generated by twisting the torsion bar. However, unlike a typical skateboard, it has two casters, so the board will tip over unless the rider continuously twists the torsion bars alternately. Specifically, this learning task requires the rider to actively propel the board themselves; otherwise, they will fall. Consequently, it is a full-body movement task that strongly encourages trial-and-error learning. Furthermore, by comparing the number of attempts required to master the task, it is relatively easy to distinguish between the learners who quickly improve and those who do not. This suggests the task aligns well with the objectives of this study.

Previous studies regarding caster boards used computer simulations (Ito et al., 2012; Agrawal et al., 2016; Gadzhiev et al., 2020) and robots (Kinugasa et al., 2013; Su et al., 2013; Wang et al., 2013; Fukai et al., 2020) to elucidate the propulsion mechanism. For example, Agúndez et al. (2021) mathematically modeled caster board propulsion and verified motion stability by manipulating variables such as propulsion speed, caster tilt angle, and torsion bar stiffness. However, Hooper (2019) noted the lack of research on human movement on caster boards. Hooper's research examined the frequency of knee flexion and extension in proficient riders and its impact on propulsion speed. Nevertheless, their study solely focused on enhancing pre-existing skills of experienced riders. Studies of the motor skills necessary for novice caster boarders to ride successfully have not been conducted. It is crucial to elucidate the motor learning process underlying behavioral changes as the learners acquire caster board skills.

Accordingly, this study investigates the learning process of beginners as they attempt to ride a caster board until they can do successfully. Specifically, the study aims to examine learners' movement changes from a dynamical systems perspective, identify factors contributing to individual differences in learning rate and acquired coordinative structures, and highlight common movement characteristics that clarify the motor skills essential for mastering the caster board.

## 2 Materials and methods

### 2.1 Participants

Seven healthy men participated in the study. Their mean age was 22.7 years (*SD*= 3.25), mean height was 1.68 m (*SD*= 0.49), and mean weight was 60.3 kg (*SD* = 6.9). All participants had no prior experience with caster boarding or similar activities, such as skateboarding or snowboarding. They were informed of the experimental procedures and provided written consent before the study began. The study protocol was approved by the Ryukoku University Ethics Committee.

### 2.2 Task and experimental conditions

**Figure 1:**
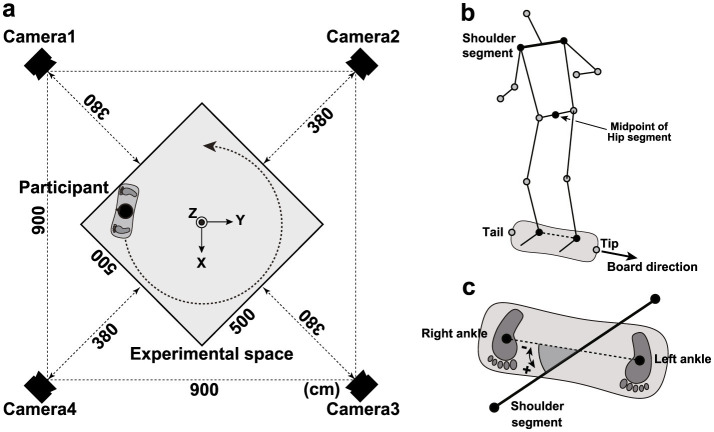
Experimental setup and variable definitions. **(a)** Location of the “measurement space” and motion-capture cameras. **(b)** Straight line connecting the shoulder segment and external capsule on the left and right sides. **(c)** Rotation angle of the shoulder segment.

The learning task required participants to complete two circular laps of a 5 m × 5 m area without falling from the caster board (Figure 1a). Participants practiced freely until they could complete both laps, maintaining a left-foot-forward stance throughout the practice period. Four motion-capture cameras (OptiTrack Flex 3; NaturalPoint, Inc., Corvallis, OR, USA) were used to record participant movements during the learning process, with a sampling frequency of 100 Hz. Reflective markers (ϕ: 20 mm) were attached to the head, left and right acromial processes, both greater trochanters, both knee joints, both ankle joints, and the tip and tail of the caster board (Figure 1b). A right-handed orthogonal reference frame was used, defined by *X*–, *Y*–, and *Z*-axes. The *Z*-axis was vertical; the *X*-axis was horizontal and directed along the diagonal. The *Y*-axis was perpendicular to the other two axes.

### 2.3 Procedure

Prior to the experiment, the experimenter demonstrated caster board riding and presented the learning task, without providing verbal instructions. However, participants were allowed to observe the demonstration as often as they wished, including during practice. In the initial stages of learning, participants struggled to place both feet on the board without assistance; thus, a chair was provided as an aid when needed.

### 2.4 Data analysis

A second-order Butterworth low-pass filter (cut-off frequency range: 6–11 Hz) was used to remove high-frequency noise from the time series data for each reflective marker. The cut-off frequency range was determined using the residual analysis method (Winter et al., 1974; Winter, 1990).

The start of each trial was defined as the point at which the participant removed his hands from the chair or placed both feet on the decks (if the chair was not used). The end of the trial was defined as the point at which either foot touched the ground. Trials were excluded from analysis if the board advanced less than half its length (39 cm) from the starting position. This approach eliminated the instances in which the participant fell from the board immediately after the start of the trial.

**Figure 2:**
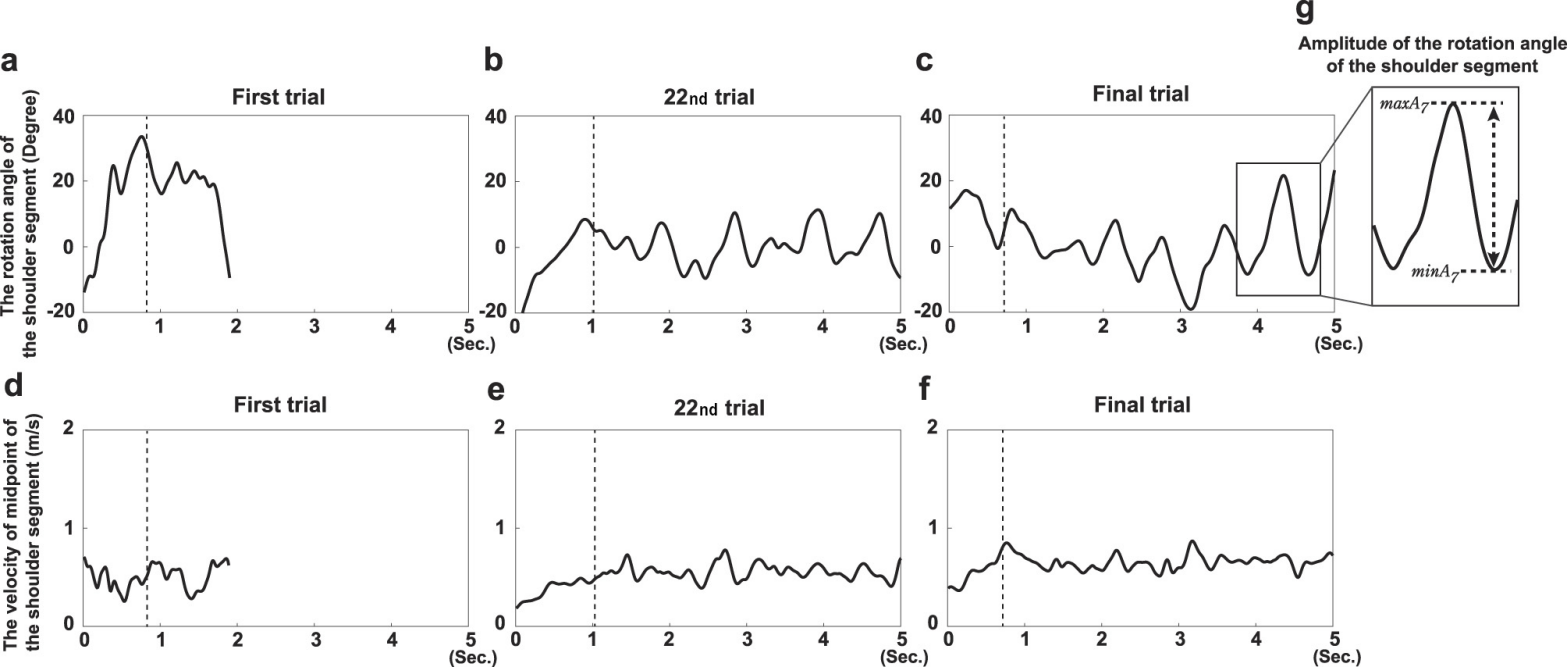
Example data obtained during the learning process for participant D. Example time series data of each variable of interest obtained over 5s in trial 1 **(a, d)**, trial 22 **(b, e)**, and the final trial **(c, f)**: the rotation angle of the shoulder segment (upper panel) and velocity at the midpoint of the hip segment (lower panel) are shown. **(g)** The process of calculating the amplitude of the shoulder segment rotation angle.

Two variables were analyzed to assess movement changes throughout the experiment. The first variable was the amplitude of trunk rotation (*A*_*TR*_). Fukai et al. (2020) examined the mechanism by which a caster board is propelled using a humanoid robot; they found that the rotational motion of the torso is important for torsion bar twisting. To propel the caster board forward, participants must twist the torsion bar by alternately applying force with the left and right legs against the respective decks. During the measurement, participants mainly performed rotational movements of the torso to achieve torsion; *A*_*TR*_ was quantified by calculating the angle between the segment connecting the left and right acromion and the segment connecting the left and right ankle joints (Figure 1c). Figures 2a–c illustrates the time series of the shoulder segment rotation angle for participant D in each of three experimental trials (trial 1, trial 22, and the final trial). The rotational angle showed periodic, rhythmic motion with amplitude increasing over the course of practice. In this time series, the amplitude was calculated by subtracting the minimum value from the maximum value for each trial (Figure 2g), with the average amplitude defined as *A*_*TR*_. Specifically, *A*_*TR*_ was calculated by the following equation.


$$\begin{array}{l} A_{TR}=\frac{\overset{N}{\underset{i=1}{\sum }}maxA_{i}-minA_{i}}{N} \end{array}$$ (1)

*N* is the number of cycles observed in each trial, *i* is the order of the cycle, and *maxA*_*i*_ and *minA*_*i*_ are the maximum and minimum angles in each cycle, respectively. Figure 2g illustrates the process of calculating the amplitude of the seventh cycle in this trial.

The second variable analyzed was the initial velocity of the board (*V*_*I*_). Agúndez et al. (2021) used computer simulations to examine variables that contribute to caster board stability; they reported that the stability increased with velocity. Thus, to maintain balance on a board only with two front and rear wheels on casters, the board must be propelled at a specific velocity. However, in actual caster boarding, this torso twisting is often performed while the board is propelled at a specific velocity after the boarder has begun to ride (i.e., by pushing the riding aids out with their hands or kicking the ground with their feet from a stationary position). Twisting the torsion bar while stationary does not produce sufficient velocity to stabilize the board, causing loss of balance. Therefore, sufficient initial velocity to stabilize the board is needed to gain thrust. Based on the previous findings, *V*_*I*_ was included as a variable in this study. However, markers placed at the leading and trailing edges of the board move perpendicularly to the board's direction of motion due to deck twisting. Therefore, the velocity of the midpoint of the hip segment was used to represent *V*_*I*_ in the direction of travel. The velocity of the midpoint of the hip segment was obtained by calculating the distance traveled by the midpoint over time. The lower panel of Figure 2 shows the time series of the velocity of the midpoint of the hip segment for participant D across three experimental trials (trial 1, trial 22, and the final trial). The midpoint velocity increased throughout the learning period. The dashed line in the figure indicates the point, *N*, at which the distance traveled by the midpoint of the hip segment from the start of the trial exceeded half the length of the board. The average velocity up to this point is *V*_*I*_. Specifically, *V*_*I*_ was calculated by the following equation.


$$\begin{array}{l} V_{I}=\frac{\overset{N}{\underset{i=1}{\sum }}V_{i}}{N} \end{array}$$ (2)

*N* represents the point mentioned above, and *V*_*i*_ represents the velocity at each time point.

#### 2.4.1 Correlation analysis between boarding performance and variables of interest

For each participant, the correlation coefficient (*r*) and effect size (*R*_2_) were calculated between the distance traveled by the board and the *A*_*TR*_ for each trial. Pearson's product–rate correlation coefficient was used to assess the correlation coefficients, and the effect size was calculated by squaring the correlation coefficient. The significance level (α) was set at 0.05, and confidence intervals (CI) were calculated. The same analysis as above was conducted for the distance traveled by the board per trial and for *V*_*I*_. This analysis aimed to examine the relationships between these variables and boarding performance.

#### 2.4.2 Learning strategy analysis

**Figure 3:**
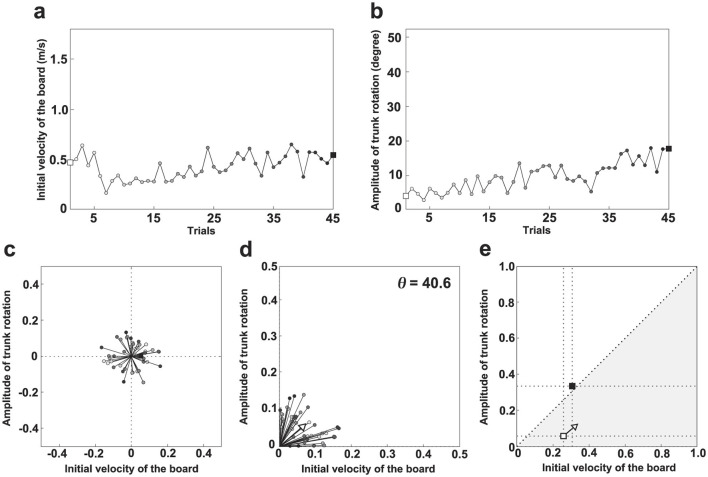
Learning strategy analysis procedure. **(a)** Trial-to-trial variation in the initial board velocity (*V*_*I*_) for participant D. **(b)** Trial-to-trial variation in the magnitude of trunk rotation (*A*_*TR*_) for participant D. **(c)** Polar coordinates of vectors based on the difference between two consecutive trials. **(d)** Polar coordinates of vectors based on the absolute difference between two consecutive trials. **(e)** First trial (□), final trial (■), and learning strategy.

The learning strategies of each participant were examined through the following analysis. Figure 3 illustrates the analysis process. Figures 3a, b show the changes in *A*_*TR*_ and *V*_*I*_ for participant D. White and black squares indicate the first and final trials, respectively. Other trials are represented by progressively darker gray squares. Figure 3c demonstrates the quantification of change in each variable between consecutive trials. The difference in values was calculated and plotted as a vector in a polar coordinate system. To account for unit differences, the highest value for each variable was normalized to 1 and the lowest to 0 across all trials and participants. In the first quadrant of the figure, values of both variables increase for a given trial; in the second quadrant, only *A*_*TR*_ increases, whereas in the third quadrant, both variables decrease. Finally, only *V*_*I*_ increases in the fourth quadrant. Figure 3d shows the absolute values of the changes expressed in Figure 3c. The arrows indicate the average vector calculated from these values. Notably, direction and magnitude of the vector (the arrow in Figures 3d, e) was calculated by the following equation.


$$\begin{array}{l} \text{Direction of vector}=\\ tan^{-1}\left(\frac{\overset{N-1}{\underset{i=1}{\sum }}|A_{TR\left(i+1\right)}-A_{TR\left(i\right)}|}{N-1}/\frac{\overset{N-1}{\underset{i=1}{\sum }}|V_{I\left(i+1\right)}-V_{I\left(i\right)}|}{N-1}\right)\\ \times 180/\pi \end{array}$$ (3)

$$\begin{array}{l} \text{Magnitude of vector}=\\ \frac{\overset{N-1}{\underset{i=1}{\sum }}\sqrt{|A_{TR\left(i+1\right)}-A_{TR\left(i\right)}{|}^{2}+|V_{I\left(i+1\right)}-V_{I\left(i\right)}{|}^{2}}}{N-1} \end{array}$$ (4)

*N* represents the number of trials for each participant, *i* is the order of the trial, and *A*_*TR*(*i*)_ and *V*_*I*(*i*)_ represent the *A*_*TR*_ and *V*_*I*_ for each trial, respectively. A steeper direction above 45 degrees suggests a greater change in *A*_*TR*_ through learning, whereas a shallower direction below 45 degrees indicates a greater change in *V*_*I*_. For participant D, the direction of the vector (Θ) was 40.6 degrees, indicating that *V*_*I*_ tended to change more than *A*_*TR*_ through learning. The direction of this vector represents the learning path indicating which of the two variables changed more. Conversely, the larger the magnitude of this vector, the greater the change in the value of both variables between the one previous trial and the current trial. Specifically, the magnitude of this vector represents the degree of the variation of the movement patterns in trial-to-trial carried out in learning process. In this study, the learning strategy was examined based on the slope and length of the vector. From the viewpoint of dynamical systems, this learning strategy corresponds to a vector field, because the direction of the vector orients the change in trajectory and the length of the vector characterizes the degree of the change in trajectory. Thus, the learner is regarded as a dynamical system, the movement pattern at the start of learning can be considered the initial state, and the coordinative structure achieved upon task completion can be regarded as the attractor. In this framework, the direction and magnitude of the vector correspond, respectively, to the learning path and the variation in movement patterns across trials during the transition from the initial state to the attractor.

Figure 3e illustrates the learning strategy between the first and final trials for participant D. The gray area indicates an instance where *V*_*I*_ is large relative to *A*_*TR*_. White and black squares represent the first (initial state) and final (attractor) trials, respectively; the learning strategy (vector field) originates from the first trial. The initial state of participant D can be characterized as *V*_*I*_-dominant, the vector field as *V*_*I*_-dominant, and the attractor as *A*_*TR*_-dominant. This analysis method enabled examination of both overall trends and individual differences in the learning process.

## 3 Results

### 3.1 Task performance

The number of trials required to complete the learning task significantly varied among participants: A (11), B (18), C (21), D (49), E (58), F (98), and G (138). However, as mentioned above, we excluded trials in which the participants fell from the board immediately after the start of the trial. Thus, the, numbers of trials analyzed as follows for each participant: A (8, 73%), B (17, 94%), C (16, 76%), D (45, 92%), E (56, 97%), F (85, 87%), and G (135, 98%). During these trials, participants A, C, D, and E required no additional demonstrations, B and F required one additional demonstration, and G required four additional demonstrations. These results indicate that the number of trials required to accomplish the task greatly varied among the learners.

**Figure 4:**
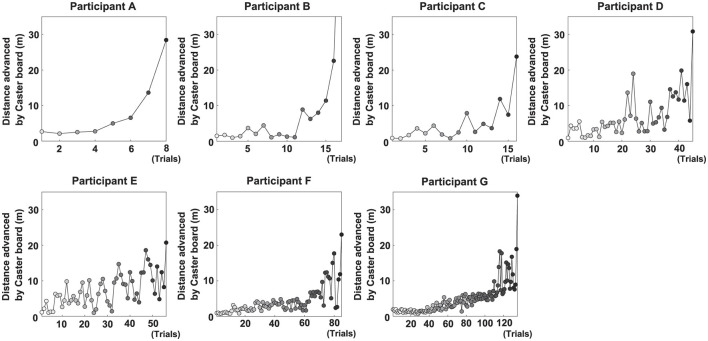
Performance trends for each participant. Each point in a given panel represents the distance traveled by the midpoint of the hip segment in a single trial.

Figure 4 shows the change in distance traveled by the midpoint of the hip segment for all participants and trials. Despite trial-to-trial variations, the distance traveled generally increased according to the number of trials.

### 3.2 Changes in variables of interest with learning

**Figure 5:**
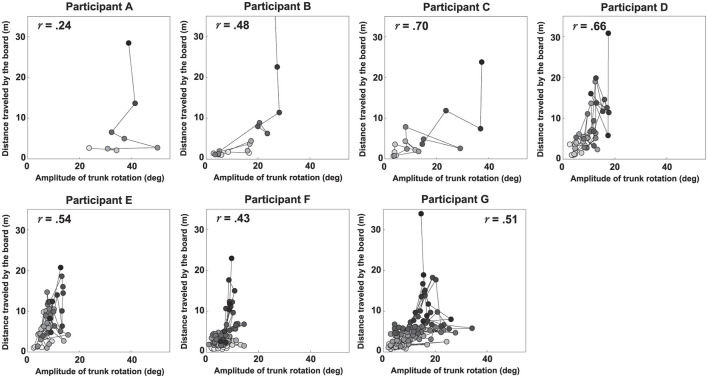
Scatter plots of the distance traveled by the board vs. the magnitude of trunk rotation for each participant. The *r* value in each panel represents the correlation coefficient.

**Figure 6:**
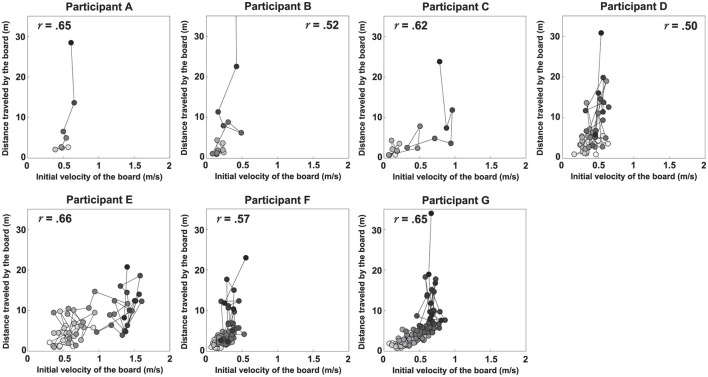
Scatter plots of the distance traveled by the board vs. its initial velocity for each participant. The *r* value in each panel represents the correlation coefficient.

**Figure 7:**
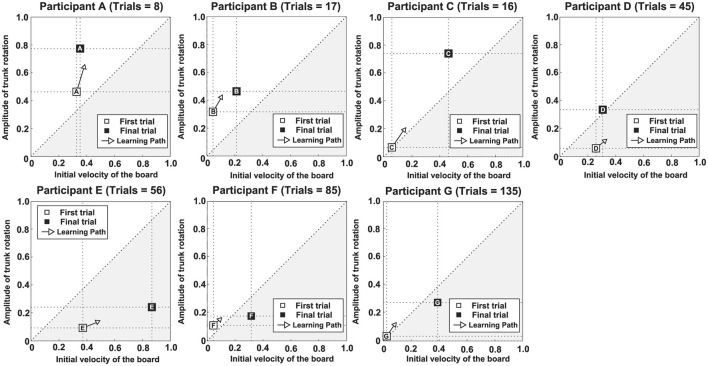
Results of learning strategy analysis for each participant. The white square (□) in each panel represents the first trial, and the black square (■) represents the final trial. Arrow direction indicates which of the two variables of interest exhibited greater change, and arrow length represents the degree of change.

Figures 5, 6 show scatter plots of board travel distance vs. *A*_*TR*_ and *V*_*I*_, respectively, for each participant. In the figure, *r* is the correlation coefficient between board travel distance and each variable; a higher value of *r* indicates a stronger correlation. Table 1 summarizes the results related to the correlation analysis. Figure 7 shows the results of the learning strategy analysis for each participant. In each figure, white and black squares represent the first and final trials, respectively. Arrow direction indicates which of the two variables showed greater change, and arrow length represents the degree of change. Table 2 summarizes these results.

**Table 1:** Results of correlation analysis between boarding performance and variables of interest.

**Participant**	**Trials**	***A***_*****TR*****_			***V***_*****I*****_		
		*r*	*R*_2_	**95% CI**	*r*	*R*_2_	**95% CI**
A	8	0.24	0.06	[–0.56, 0.81]	0.65	0.42	[–0.10, 0.93]
B	17	0.48	0.23	[0.00, 0.78]	0.52	0.27	[0.05, 0.80]
C	16	0.70	0.49	[0.31, 0.89]	0.62	0.38	[0.18, 0.85]
D	45	0.66	0.44	[0.45, 0.80]	0.50	0.25	[0.24, 0.69]
E	56	0.54	0.29	[0.32, 0.70]	0.66	0.44	[0.48, 0.79]
F	85	0.43	0.18	[0.37, 0.63]	0.57	0.32	[0.41, 0.70]
G	135	0.51	0.26	[0.37, 0.63]	0.65	0.42	[0.54, 0.74]

**Table 2:** Results of learning strategy analysis.

**Participant**	**Trials**	**First trial**			**Vector of leaning path**			**Final trial**		
		*A*_*TR*_	*V*_*I*_	**Dominance**	**Direction**	**Dominance**	**Magnitude**	*A*_*TR*_	*V*_*I*_	**Dominance**
A	8	0.46	0.33	*A*_*TR*_	73.1	*A*_*TR*_	0.19	0.77	0.35	*A*_*TR*_
B	17	0.32	0.04	*A*_*TR*_	59.4	*A*_*TR*_	0.12	0.46	0.21	*A*_*TR*_
C	16	0.07	0.06	*A*_*TR*_	55.5	*A*_*TR*_	0.15	0.74	0.47	*A*_*TR*_
D	45	0.06	0.26	*V*_*I*_	40.6	*V*_*I*_	0.09	0.34	0.31	*A*_*TR*_
E	56	0.09	0.37	*V*_*I*_	21.2	*V*_*I*_	0.12	0.24	0.87	*V*_*I*_
F	85	0.11	0.04	*A*_*TR*_	43.1	*V*_*I*_	0.07	0.18	0.32	*V*_*I*_
G	135	0.03	0.02	*A*_*TR*_	55.5	*A*_*TR*_	00.11	0.28	0.39	*V*_*I*_

Among all participants, Participant A had the lowest number of trials to complete the task (eight). This participant exhibited a high *A*_*TR*_ of 0.46 and a high *V*_*I*_ of 0.33 in the first trial; his *A*_*TR*_ was highest among all participants (Figure 7 and Table 2). Learning strategy analysis revealed that this participant also had the highest vector direction (73.1) and magnitude (0.19) values. These results suggested a strong tendency for the torso rotation movement to change with learning; they also suggested large movement variability within each trial. In the final trial, *A*_*TR*_ improved to 0.77, and the rotational movement of the torso was greatest. However, the correlation between board travel distance and *A*_*TR*_ was low (0.24, Figure 5), possibly because the amount of torso rotation was lower during the latter trials. In summary, this participant was characterized as having achieved an *A*_*TR*_-dominant attractor from an *A*_*TR*_-dominant initial state large where both variables were large, through a strong *A*_*TR*_-oriented vector field.

Participant B required 17 trials to complete the task. In the first trial, *A*_*TR*_ was higher than *V*_*I*_ (0.32 and 0.04, respectively); this trend persisted in the final trial (0.46 and 0.21, respectively). These results suggested that board propulsion relied more on trunk twisting than on initial *V*_*I*_. Learning strategy analysis also revealed a greater change in *A*_*TR*_ during the learning process, indicated by a vector direction and magnitude value of 59.4 and 0.12 (Figure 7 and Table 2). The correlation coefficient between board travel distance and *A*_*TR*_ was 0.48, and the correlation coefficient for *V*_*I*_ was similar (0.52). Overall, this participant was characterized as having achieved an *A*_*TR*_-dominant attractor from an *A*_*TR*_-dominant initial state through an *A*_*TR*_-oriented vector field.

Participant C required 16 trials to complete the task, the second lowest number among all participants. The initial *A*_*TR*_ was 0.07 and initial *V*_*I*_ was 0.06; these values were lower than those of the previous two participants. However, values in the final trial substantially increased (*A*_*TR*_: 0.74, *V*_*I*_: 0.47). Learning strategy analysis showed that the vector direction value was 55.5, indicating a greater change in the rotational movement of the torso. The vector magnitude (0.15) was the second longest among all participants, suggesting high movement variability in each trial. The correlation coefficient between board travel distance and *A*_*TR*_ was 0.70, and the correlation coefficient for *V*_*I*_ was similar (0.62). In summary, this participant transitioned from an *A*_*TR*_-dominant initial state, in which both variables were small, through a strong vector field oriented toward *A*_*TR*_, to an *A*_*TR*_-dominant attractor with markedly improved *A*_*TR*_ and *V*_*I*_.

Participant D required 45 trials to complete the task. In the first trial, *V*_*I*_ was higher than *A*_*TR*_ (0.26 and 0.06, respectively), and the initial velocity of the board was the dominant variable. In the final trial, the trend was reversed (*V*_*I*_, 0.31; *A*_*TR*_, 0.34), but the value of *A*_*TR*_ was smaller than for the previous participants. The vector direction value was 40.6 and its magnitude was 0.09, indicating that *V*_*I*_ had substantially changed. The correlation coefficients of board travel distance with *A*_*TR*_ and *V*_*I*_ were 0.66 and 0.50, respectively. In summary, this participant was characterized as having achieved an *A*_*TR*_-dominant attractor from a *V*_*I*_-dominant initial state through a *V*_*I*_-oriented vector field.

Participant E required 56 trials to complete the task. In the first trial, *V*_*I*_ was much higher than *A*_*TR*_ (0.37 and 0.09, respectively); this trend persisted in the final trial (*V*_*I*_, 0.87; *A*_*TR*_, 0.24). These findings suggest that this participant tended to propel the board by increasing *V*_*I*_ rather than performing rotational movement of the torso. Learning strategy analysis suggested that *V*_*I*_ considerably varied, with a vector direction and magnitude value of 21.2 and 0.12. The correlation coefficients of the board travel distance with *A*_*TR*_ and *V*_*I*_ were 0.54 and 0.66, respectively. From the above, this participant was characterized as having achieved a *V*_*I*_-dominant attractor from a *V*_*I*_-dominant initial state through a *V*_*I*_-oriented vector field.

Participant F required 85 trials to complete the task, the second highest number among all participants. In the first trial, *V*_*I*_ was higher than *A*_*TR*_ (0.04 and 0.11, respectively), although *A*_*TR*_ was smaller than the values of participants A and B, who had similar trends. In the final trial, the difference in values disappeared (*A*_*TR*_, 0.18; *V*_*I*_, 0.32), and *A*_*TR*_ was lowest among all participants for this trial. The vector had a direction value of 43.1 and a magnitude of 0.07, indicating that although *V*_*I*_ significantly changed, the length value was lowest among all participants for this trial. The correlation coefficients of the board travel distance with *A*_*TR*_ and *V*_*I*_ were 0.43 and 0.57, respectively. Overall, this participant was characterized as having achieved a *V*_*I*_-dominant attractor from an *A*_*TR*_-dominant initial state through a *V*_*I*_-oriented vector field.

Participant G required the most trials to complete the task (135 trials) among all participants. Initial *A*_*TR*_ and *V*_*I*_ values were lowest among all participants (0.03 and 0.02, respectively). In the final trial, *V*_*I*_ was the dominant variable (*A*_*TR*_, 0.28; *V*_*I*_, 0.39). Learning strategy analysis yielded a vector direction value of 55.5 and a magnitude of 0.11, indicating a strong tendency to change the rotational motion of the torso. The correlation coefficients of the board travel distance with *A*_*TR*_ and *V*_*I*_ were 0.51 and 0.65, respectively. In summary, this participant was characterized as having achieved a *V*_*I*_-dominant attractor from an initial state of *A*_*TR*_ dominance, where both variables were small, through an *A*_*TR*_-oriented vector field.

These results indicate that caster board performance improved with each successive trial for all participants. Furthermore, the values of *A*_*TR*_ and *V*_*I*_ on the final trial increased compared to the first trial (Table 2). However, the number of trials required to accomplish the task varied across participants, and individual differences were observed in the values of both variables in the first and final trials, as well as in the learning strategies. Factors contributing to these results are discussed in the following section.

## 4 Discussion

This study observed the learning process of seven participants attempting to ride a caster board for the first time, tracking their progress until they could successfully ride. Changes in the learning process were compared across two variables: initial board velocity and the amplitude of trunk rotation. The study specifically aimed to clarify the factors underlying individual differences in learning by examining changes in movement patterns from the first trial to the coordinative structure achieved in the final trial, as well as the learning strategies employed. Additionally, we examined key motor skills for mastering the caster board by focusing on commonalities observed among individual participants.

First, we discuss the key motor skills required to master the caster board. Results from Figures 5, 6 and Table 1 reveal that, across all participants, both variables *V*_*I*_ and *A*_*TR*_ generally increased as the distance traveled increased. This indicates that to ride the caster board skillfully, it is necessary to increase the board's initial velocity and to further apply propulsive force to the board by performing rotational movement of the torso. These results are consistent with previous studies (Fukai et al., 2020; Agúndez et al., 2021) that identifies key factors for caster board riding via computer simulations and robotics.

However, the learning processes of individual participants suggest caution in concluding that merely improving *A*_*TR*_ and *V*_*I*_ is sufficient for a learner to ride a caster board. For example, in the learning process for bicycles—which, such as the caster board, is a two-wheeled vehicle—the first critical step is to maintain balance while moving at a certain speed. Only afterward does one typically learn the pedaling motion to generate forward momentum (Mercê et al., 2024). The results of this study also indicate that participants D through G reached the task goal despite having low *A*_*TR*_ values (Table 2). This suggests that maintaining sufficient board propulsion velocity is a necessary condition for mastering the caster board. Furthermore, it is inferred that mastering trunk rotational movement is required as the action to generate the board's propulsive force. During participant A's learning process, *A*_*TR*_ peaked four trials before the final trial, then declined and never reached that peak again, including in the final trial (Figure 5). This suggests that excessive trunk rotation on a two-wheeled caster board can also be a factor in losing balance.

As mentioned above, it has been established that the board's initial velocity and torso rotation are crucial for stable riding on a caster board. However, as noted by Hooper (2019), the frequency of knee flexion and extension also influences propulsion speed. Possibly, as riders become more skilled, their coordinative structures shift—reducing reliance on torso rotation and generating propulsion primarily through lower-body movement. Thus, the motor skills required for stable riding may differ from those needed for more advanced performance. Given that this study did not investigate the learning process beyond the point at which participants were able to ride the caster board, it does not reveal the complete range of motor skills involved. To achieve this, it is thought that observing movements during more advanced motor tasks and comparing them with expert movements will be necessary. Specifically, by observing movements in situations where greater board propulsion is required for stable riding—such as zigzagging, riding at higher speeds, or ascending slopes—the specific motor skills necessary to generate this propulsion can be more precisely identified.

Next, we address the factors contributing to individual differences in the learning process. As shown in Table 2, even when the learning task was identical, individual differences were observed in the final learning outcomes—that is, the coordinative structure achieved upon task completion and the learning rate. The factors underlying these differences, such as the movement patterns during the first trial and the learning strategies employed, varied considerably among participants.

One possible factor is the learning pathway shaped by the movement pattern in the first trial. As noted above, individual differences were observed in each participant's learning process. However, these differences were not entirely random. Correlations were also identified among several elements. Specifically, among the seven participants, five showed a match between dominance in the coordinative structure during task completion and dominance in the learning path. Furthermore, among the seven participants, six showed a match between dominance in the learning path and dominance in the movement pattern during the first trial. Moreover, four participants exhibited a match between dominance in the coordinative structure and dominance in the movement pattern during the first trial. These findings suggest that the learning path (i.e., vector direction within the vector field) influences the coordinative structure (i.e., the attractor) achieved upon task completion. They also indicate that the movement pattern during the first trial (i.e., the initial state) can shape the learning path. The above findings are also thought to be related to learning rate. Among the top four participants who required fewer trials to reach the task, the final trial showed a dominant *A*_*TR*_ coordinative structure. Furthermore, among the top three participants (participants A, B, and C) *A*_*TR*_ was dominant both in the first trial and the learning path. By contrast, among the bottom three participants, the final trial showed a dominant *V*_*I*_ coordinative structure, and for two of them, the learning path also favored *V*_*I*_. This suggests that participants A, B, and C, who showed dominant *A*_*TR*_ in the first trial and learning path, had already discovered this during the early stage of learning. Conversely, since *V*_*I*_ was the dominant learning path for participants D, E, and F, these participants may have focused on the board's velocity required for stable boarding and neglected learning the movements necessary to generate propulsive force. Thus, it became clear that the first trial's movement pattern caused learning paths to diverge, potentially influencing the coordinative structure and learning rate. This finding indirectly supports the view of Kostrubiec et al. (2012) that movement patterns performed by learners early in the learning process, which they refer to as predispositions, influence the coordinative structure achieved upon task completion, even in the learning of caster board movements.

However, some participants could not be explained solely by the aforementioned factors as causes of individual differences in the learning process. Participant G required the most trials to achieve the task despite having a dominant *A*_*TR*_ in both the first trial and the learning path. Furthermore, considering the degree of both variables in the first trial, Participant C completed the task in only 16 trials—the second fewest number of trials—despite having values for both variables comparable to those of Participant G. Additionally, there was a significant difference between the two participants in their final coordinative structures. One factor contributing to these differences may be the variation in the movement patterns performed during the learning process. The magnitude of the vector in Participant C's learning strategy was the second largest (0.15), after that of Participant A. This indicates that changes in movement patterns between consecutive trials were substantial throughout the learning process. In particular, since the board's movement distance increased significantly after the 10th trial in the performance (Figure 4), calculating the vector magnitude up to the 10th trial and the vector magnitude after the 10th trial yielded values of 0.11 and 0.25, respectively. This indicates that this participant significantly changed their movement pattern across trials after the 10th trial. Conversely, for Participant G, a significant increase in the board's distance was observed after Trial 110 (Figure 4), while no major fluctuations were seen in the trials prior to that. Particularly, the board's distance appeared stagnant in trials before trial 40. Calculating the magnitude of the vector for each of the above intervals yielded values of 0.08 for trials before trial 40, 0.13 for trials 41 to 110, and 0.11 for trials 111 to the final trial. This suggests that throughout the learning process, this participant exhibited minimal variations in movement patterns across trials, showing a particularly strong tendency to perform similar movement patterns before trial 40. Furthermore, Participant A, who required the fewest trials to complete the task, had a high vector magnitude of 0.19. By contrast, Participant F, who required the second-highest number of trials, had a low vector magnitude of 0.07 (Table 2). This pattern suggests a correlation between the final learning outcome and vector magnitude. The above findings suggest that, during the learning process, the extent to which individuals attempt movement patterns different from those in previous trials can influence both the coordinative structure and the learning rate. Thus, the ability to explore novel movement patterns may determine how learning unfolds. Variability in movement has been identified as an essential element of self-organization (Davids et al., 2008). Accordingly, exploratory behavior—actively attempting different movements rather than adhering to the current one—is considered crucial for promoting motor learning.

In conclusion, this study examined the learning process of caster board riding from the perspective of the dynamical systems approach. The findings revealed two key factors that can generate individual differences in motor learning: The learning path influenced by the movement pattern of the first trial and the variation in movement patterns across trials. However, numerous other factors have been identified as contributing to individual differences in motor learning, including cognitive and physical abilities, as well as neuroscientific and genetic factors (Ackerman, 2014; Seidler and Carson, 2017). Additionally, factors such as decreased motivation and fatigue resulting from prolonged practice sessions, as seen in participants F and G in this study, may contribute to individual differences. Given that this study only examined the learning processes of seven participants on a case-by-case basis, it is difficult to conclude that all factors contributing to individual differences in motor learning have been fully identified. Future research should classify learners prior to instruction based on relevant abilities and factors, then compare learning trajectories across groups. Such work may provide deeper insights into individual differences in motor learning.

## Data Availability

The original contributions presented in the study are included in the article/Supplementary material, further inquiries can be directed to the corresponding author.
