# Supplementary material for: Factors influencing caster board skill acquisition
Source: Front Psychol. 2025 Nov 7;16:1643100. doi: 10.3389/fpsyg.2025.1643100 (PMC12642812; doi:10.3389/fpsyg.2025.1643100)
Supplement: Supplementary file 1 [file Data_Sheet_1.pdf]

Supplementary Material

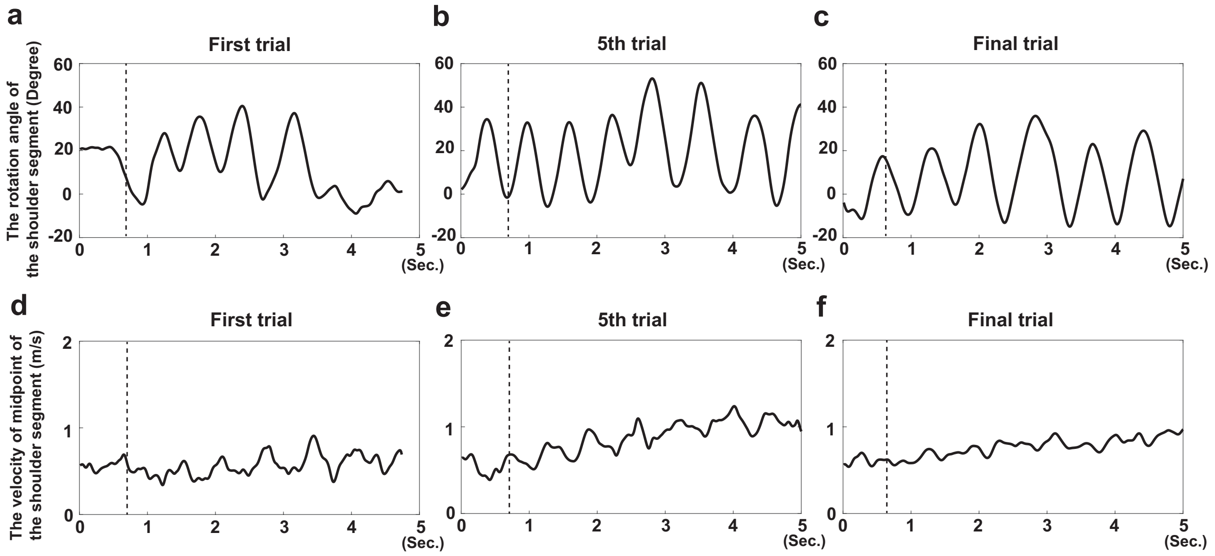

Figure S1. Example data obtained during the learning process for participant A. Example time series data of each variable of interest obtained over 5s in trial 1 (a and d), trial 5 (b and e), and the final trial (e and f): the rotation angle of the shoulder segment (upper panel) and velocity at the midpoint of the hip segment (lower panel) are shown.

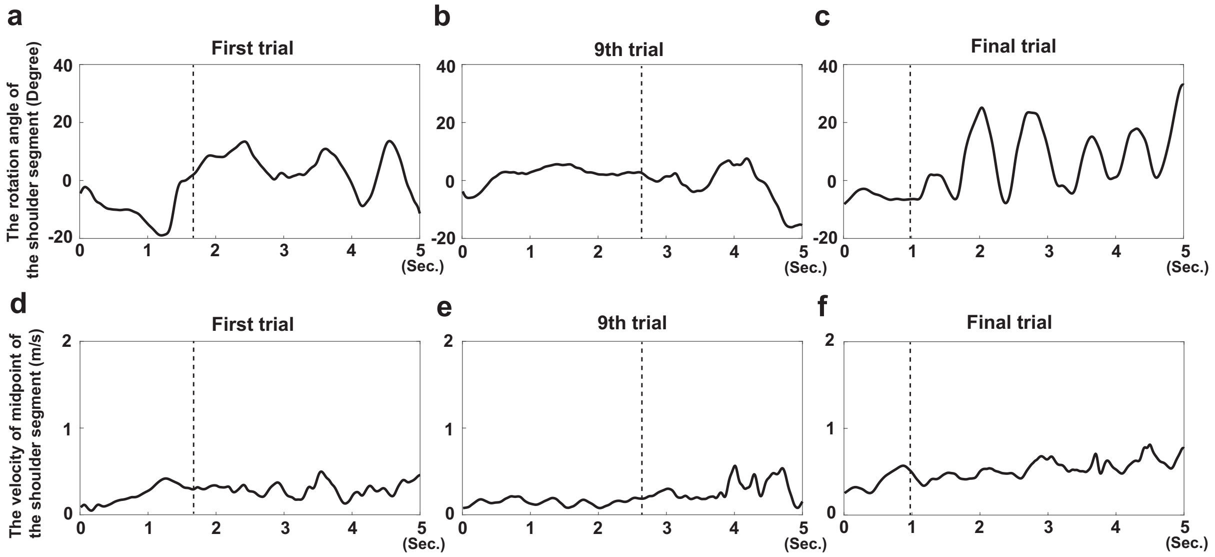

Figure S2. Example data obtained during the learning process for participant B. Example time series data of each variable of interest obtained over 5s in trial 1 (a and d), trial 9 (b and e), and the final trial (e and f): the rotation angle of the shoulder segment (upper panel) and velocity at the midpoint of the hip segment (lower panel) are shown.

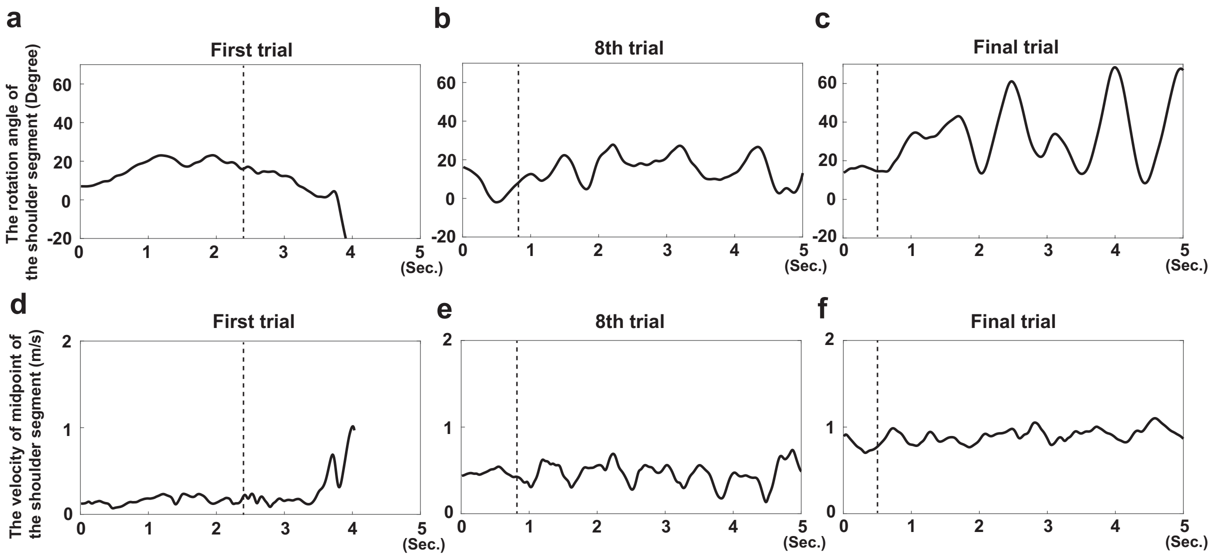

Figure S3. Example data obtained during the learning process for participant C. Example time series data of each variable of interest obtained over 5s in trial 1 (a and d), trial 8 (b and e), and the final trial (e and f): the rotation angle of the shoulder segment (upper panel) and velocity at the midpoint of the hip segment (lower panel) are shown.

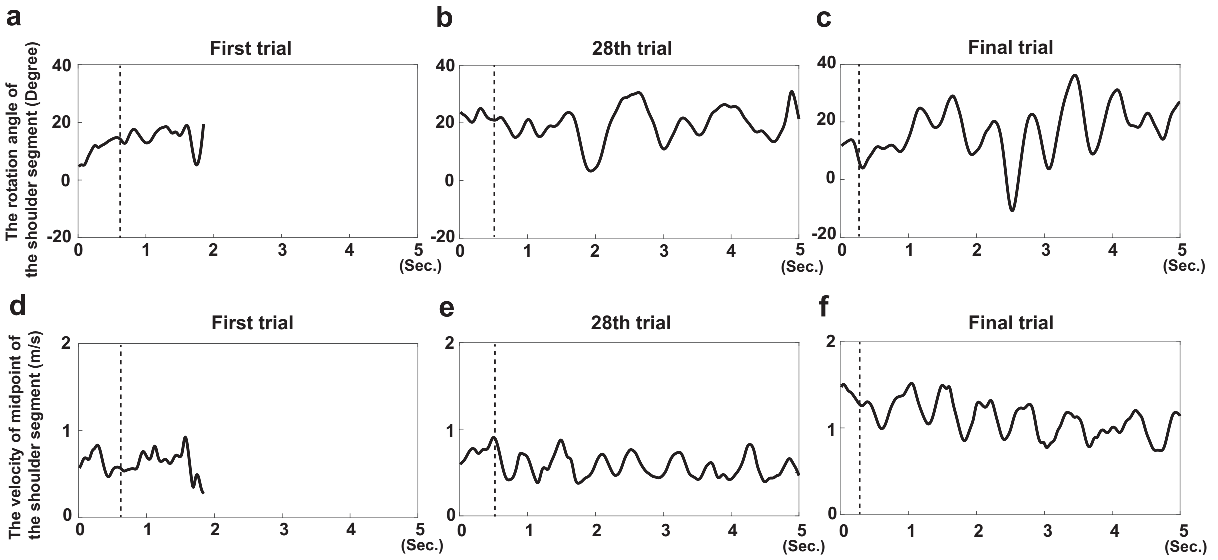

Figure S4. Example data obtained during the learning process for participant E. Example time series data of each variable of interest obtained over 5s in trial 1 (a and d), trial 28 (b and e), and the final trial (e and f): the rotation angle of the shoulder segment (upper panel) and velocity at the midpoint of the hip segment (lower panel) are shown.

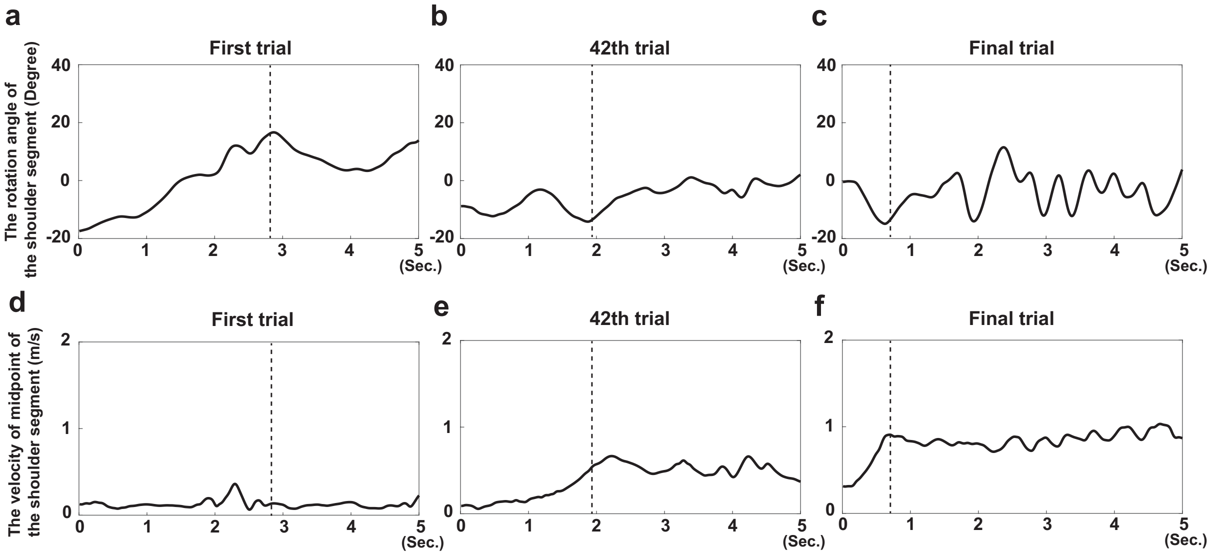

Figure S5. Example data obtained during the learning process for participant F. Example time series data of each variable of interest obtained over 5s in trial 1 (a and d), trial 42 (b and e), and the final trial (e and f): the rotation angle of the shoulder segment (upper panel) and velocity at the midpoint of the hip segment (lower panel) are shown.

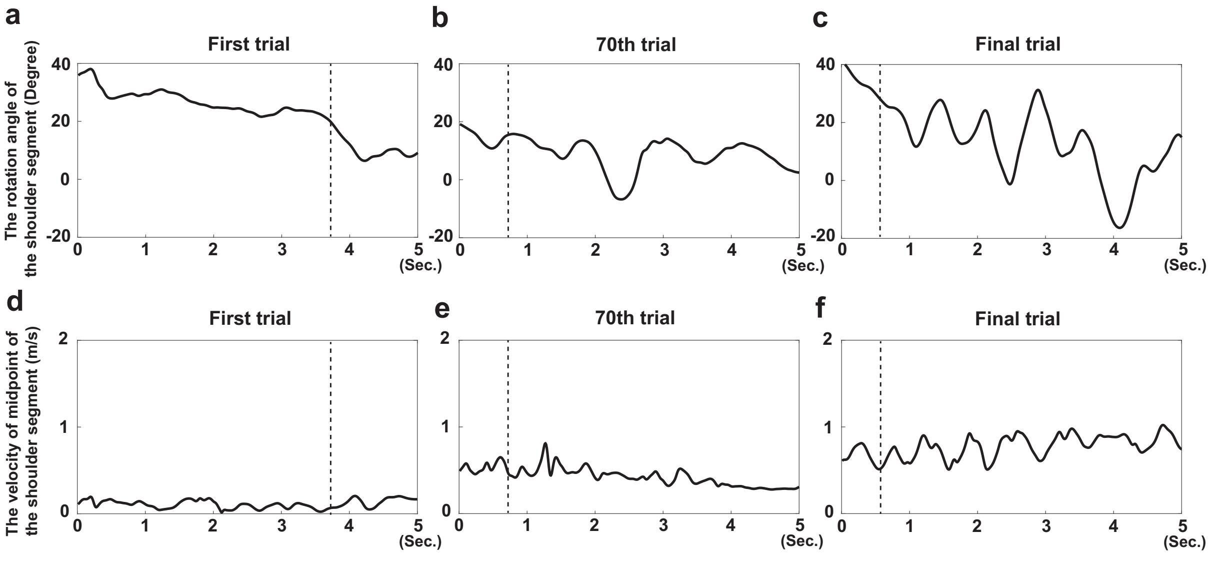

Figure S6. Example data obtained during the learning process for participant G. Example time series data of each variable of interest obtained over 5s in trial 1 (a and d), trial 70 (b and e), and the final trial (e and f): the rotation angle of the shoulder segment (upper panel) and velocity at the midpoint of the hip segment (lower panel) are shown.

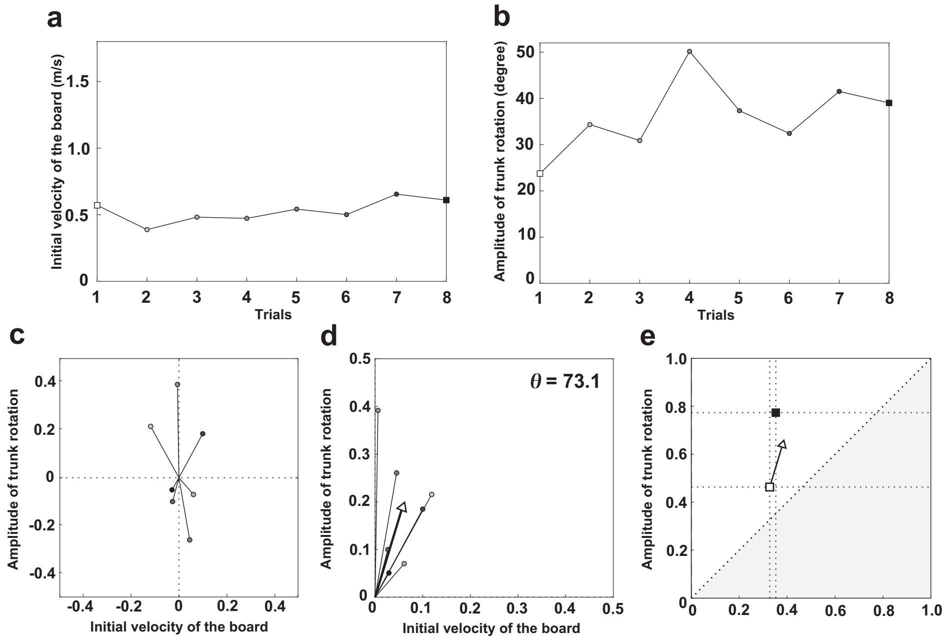

Figure S7. Learning strategy analysis procedure for participant A. (a) Trial-to-trial variation in the initial board velocity (V_I). (b) Trial-to-trial variation in the magnitude of trunk rotation (A_{TR}). (c) Polar coordinates of vectors based on the difference between two consecutive trials. (d) Polar coordinates of vectors based on the absolute difference between two consecutive trials. (e) First trial (□), final trial (■), and learning path.

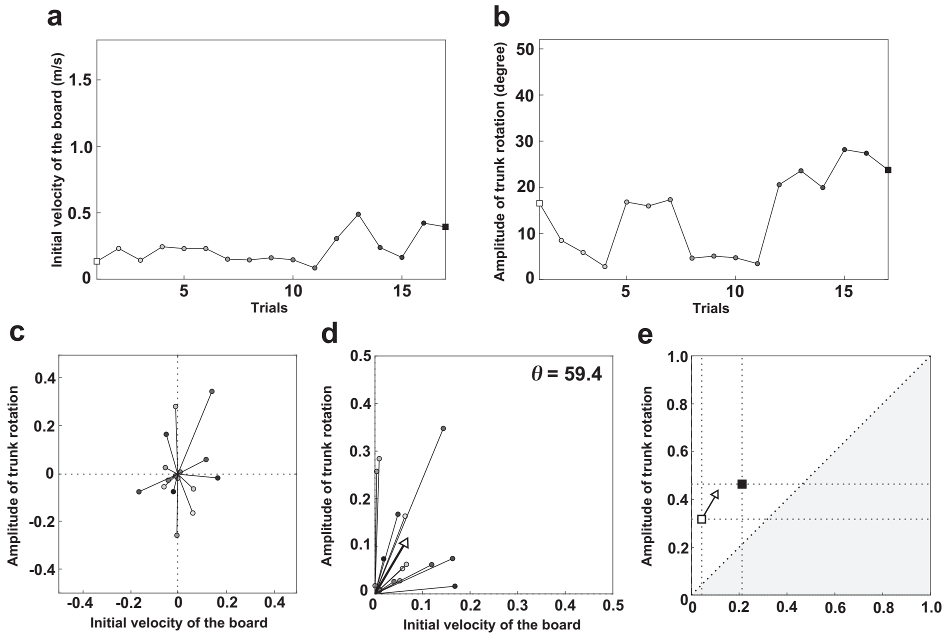

Figure S8. Learning strategy analysis procedure for participant B. (a) Trial-to-trial variation in the initial board velocity (V_I). (b) Trial-to-trial variation in the magnitude of trunk rotation (A_{TR}). (c) Polar coordinates of vectors based on the difference between two consecutive trials. (d) Polar coordinates of vectors based on the absolute difference between two consecutive trials. (e) First trial (□), final trial (■), and learning path.

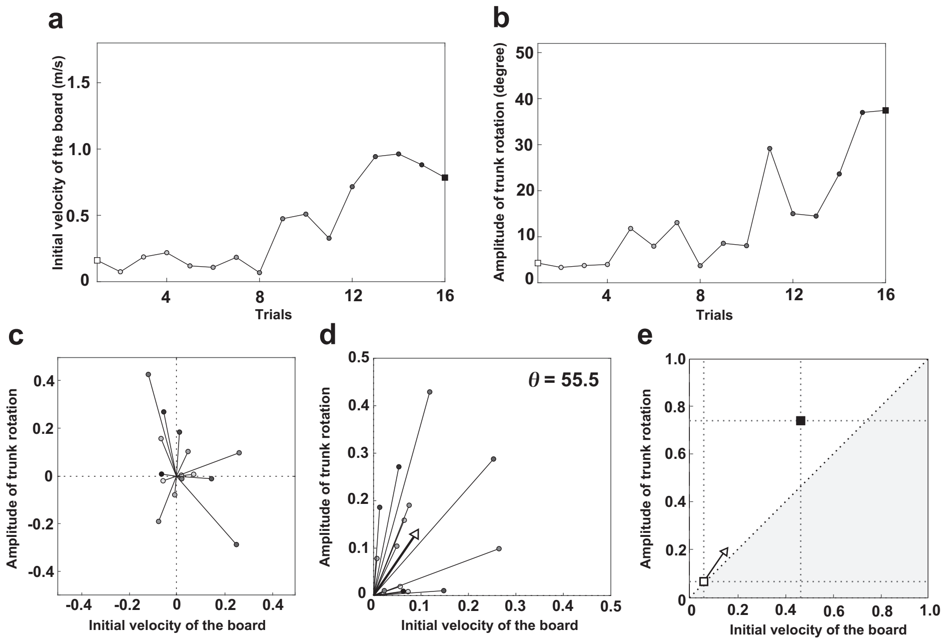

Figure S9. Learning strategy analysis procedure for participant C. (a) Trial-to-trial variation in the initial board velocity (V_I). (b) Trial-to-trial variation in the magnitude of trunk rotation (A_{TR}). (c) Polar coordinates of vectors based on the difference between two consecutive trials. (d) Polar coordinates of vectors based on the absolute difference between two consecutive trials. (e) First trial (□), final trial (■), and learning path.

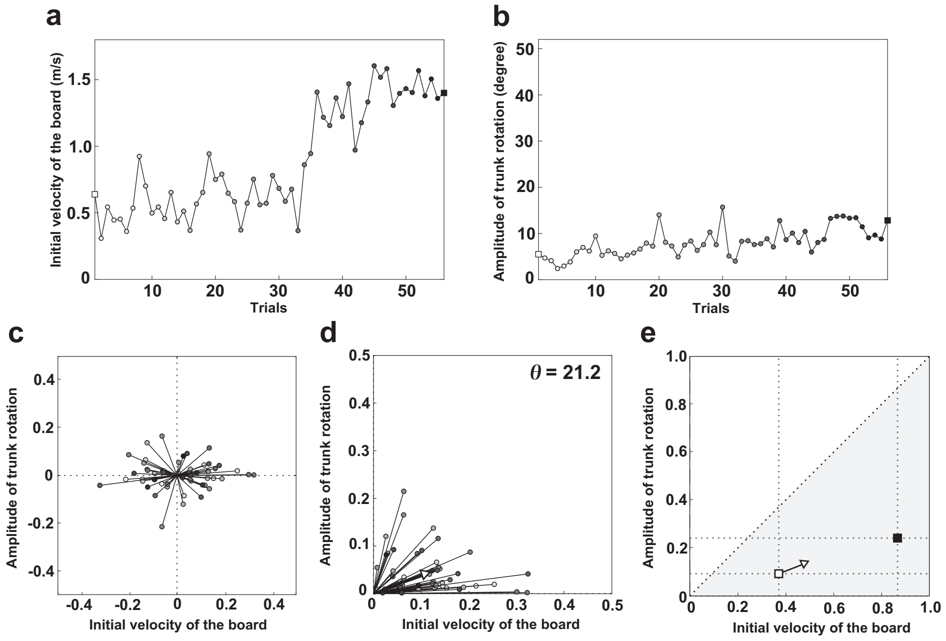

Figure S10. Learning strategy analysis procedure for participant E. (a) Trial-to-trial variation in the initial board velocity (V_I). (b) Trial-to-trial variation in the magnitude of trunk rotation (A_{TR}). (c) Polar coordinates of vectors based on the difference between two consecutive trials. (d) Polar coordinates of vectors based on the absolute difference between two consecutive trials. (e) First trial (□), final trial (■), and learning path.

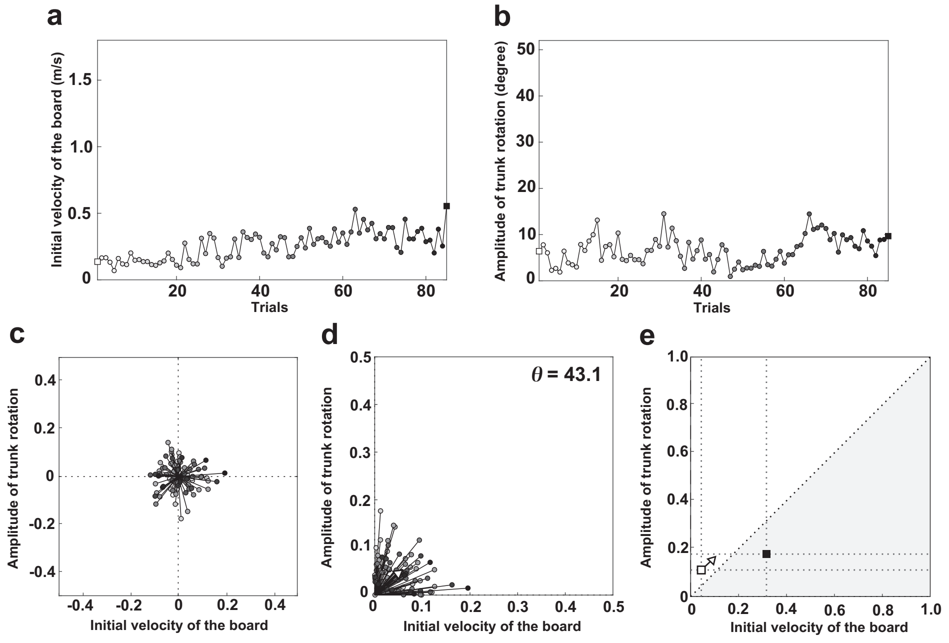

Figure S11. Learning strategy analysis procedure for participant F. (a) Trial-to-trial variation in the initial board velocity (V_I). (b) Trial-to-trial variation in the magnitude of trunk rotation (A_{TR}). (c) Polar coordinates of vectors based on the difference between two consecutive trials. (d) Polar coordinates of vectors based on the absolute difference between two consecutive trials. (e) First trial (□), final trial (■), and learning path.

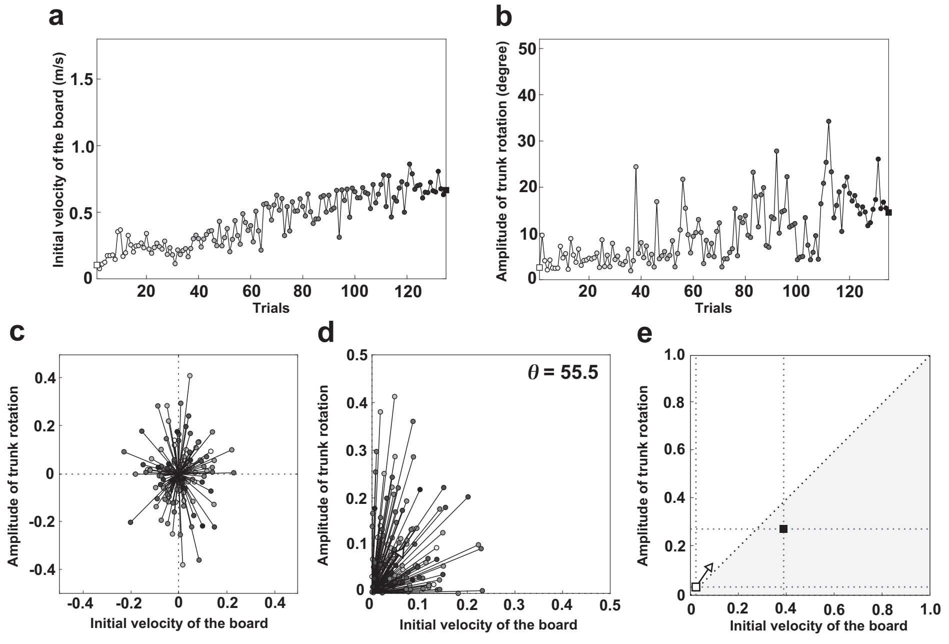

Figure S12. Learning strategy analysis procedure for participant G. (a) Trial-to-trial variation in the initial board velocity (V_I). (b) Trial-to-trial variation in the magnitude of trunk rotation (A_{TR}). (c) Polar coordinates of vectors based on the difference between two consecutive trials. (d) Polar coordinates of vectors based on the absolute difference between two consecutive trials. (e) First trial (□), final trial (■), and learning path.
